# Reply to Robinson et al.: Data integration will form the basis of future abundance estimates

**DOI:** 10.1073/pnas.2117920119

**Published:** 2022-03-01

**Authors:** Corey T. Callaghan, Shinichi Nakagawa, William K. Cornwell

**Affiliations:** ^a^School of Biological, Earth and Environmental Sciences, UNSW Sydney, Sydney, NSW 2052, Australia

We thank Robinson et al. (1) for their interest in our paper quantifying the global species abundance distribution (gSAD) of birds (2). We agree with some of their points regarding uncertainty and bias. As mentioned in the original article, uncertainty for some species is very large, and we reiterate that for many species of conservation interest there are less uncertain datasets—usually derived from structured sampling—that should be used for conservation-based decisions. We do not suggest that our estimates should be used in place of better, high-quality data. However, this local-scale, highly structured data approach cannot be scaled up to all species. Consequently, data integration is a key frontier in ecology and conservation (3), where heterogeneous data sources are used to further our understanding of population estimates. Reducing uncertainty in future abundance estimates is an important goal, and toward this end data integration methods should use all available data, such as the massive datasets being generated through citizen science initiatives. Increasing training data in our model will inevitably reduce uncertainty for many species, as highlighted by figures 4A and 4C in ref. 2 and the corresponding discussion. We also agree that there is a bias in available training data toward the developed world, which is also true of biodiversity data generally (4).

However, we disagree with some points made by Robinson et al. (1). The first is that biases are unquantifiable. Our modeling framework, as applied to the training data, was not biased as described by the analysis represented in figure S14 of ref. 2. We also disagree with the assertion that these data cannot be used for macroecological theory and empirical understanding of species abundance distributions. Our results support a rich literature that has repeatedly found log-left skew SADs (see some of the references in ref. 5). Also, independent lines of macroecological theories [e.g., Wilkinson’s “broken plate” model (6)] provide validation of our gSAD.

**Fig. 1.**
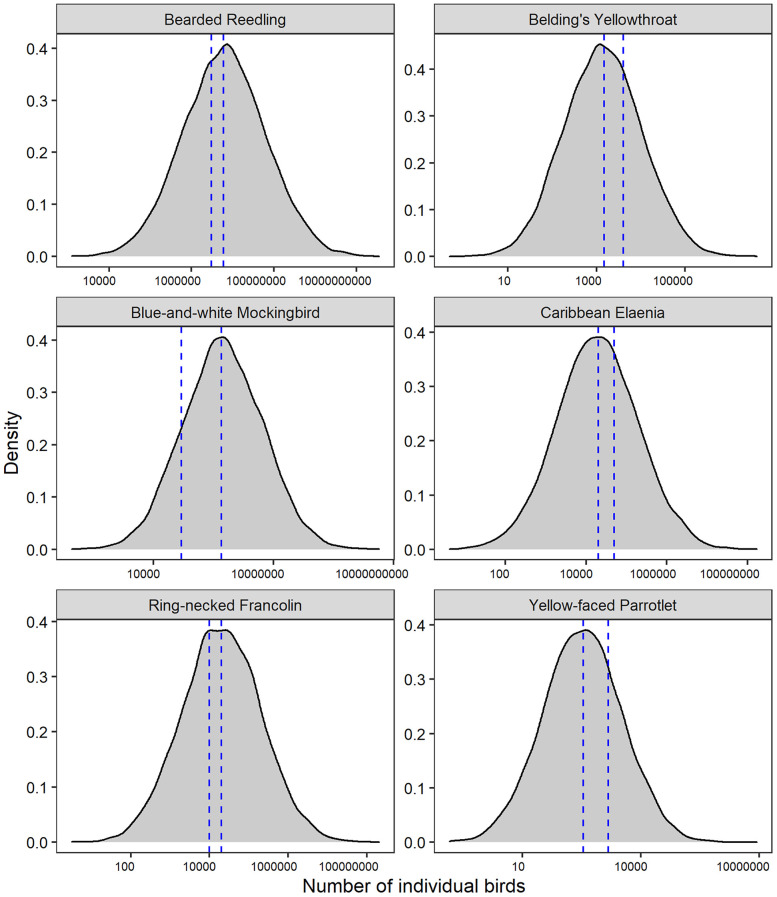
Six species that Robinson et al. (1) imply are “incorrect” as our estimate (i.e., the median of the posterior) falls outside the “minimum–maximum range” supplied by BirdLife. The blue dashed lines represent the BirdLife minimum and maximum estimates, which correspond well with our model posterior uncertainty (gray density distribution).

**Fig. 2.**
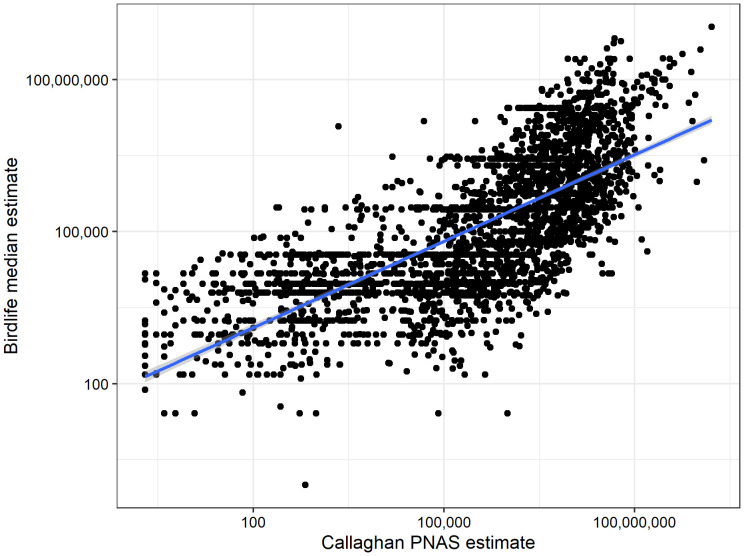
Despite the incongruencies between the datasets, and potential errors in both datasets, we find that our modeled estimates are strongly correlated with BirdLife abundance estimates (*r* = 0.72), suggesting that our method can estimate the abundances of species proportional to one another. A total of 2,860 species that corresponded between the two datasets are shown. Both axes are log10-transformed after a constant of 1 has been added. The blue line and associated gray shading represent a linear model and 95% confidence interval. An important next step, currently ongoing in our work, is to identify the species that are “outliers” in this relationship, which will help inform iterative refinements of current methods.

Robinson et al. (1) make several comparisons of our modeled estimates with a BirdLife dataset of global population estimates. However, direct comparison with the BirdLife dataset is not entirely valid as ∼25% of species in their estimates do not include uncertainty. Nevertheless, they highlight that 81% of our model estimates (i.e., median) do not fall within BirdLife “minimum–maximum ranges.” However, this assertion does not incorporate magnitude, and for many species that fall outside their minimum–maximum range our model posterior corresponds very well with the BirdLife estimates (e.g., Fig. 1). Moreover, despite the incongruencies between the datasets, our modeled estimates are strongly correlated with BirdLife abundance estimates (*r* = 0.72; Fig. 2).

Because “no method currently exists to estimate global population sizes” (1) does not mean we—as a collective community of ecologists and scientists—should not attempt to develop such methods. We see our work as the first step toward an ambitious goal of data integration using globally available citizen science data to further our understanding of abundance. Iterative refinement of methods and increasing training data will both make progress toward this ambitious goal.
